# Supplementary material for: Unilateral Tamoxifen-Induced Retinopathy as a Consequence of Breast Cancer Treatment—Multimodal Imaging Value
Source: Diagnostics (Basel). 2023 Mar 27;13(7):1250. doi: 10.3390/diagnostics13071250 (PMC10093114; doi:10.3390/diagnostics13071250)
Supplement: Supplementary file 1 [file diagnostics-13-01250-s001.zip › diagnostics-2245903-supplementary.pdf]

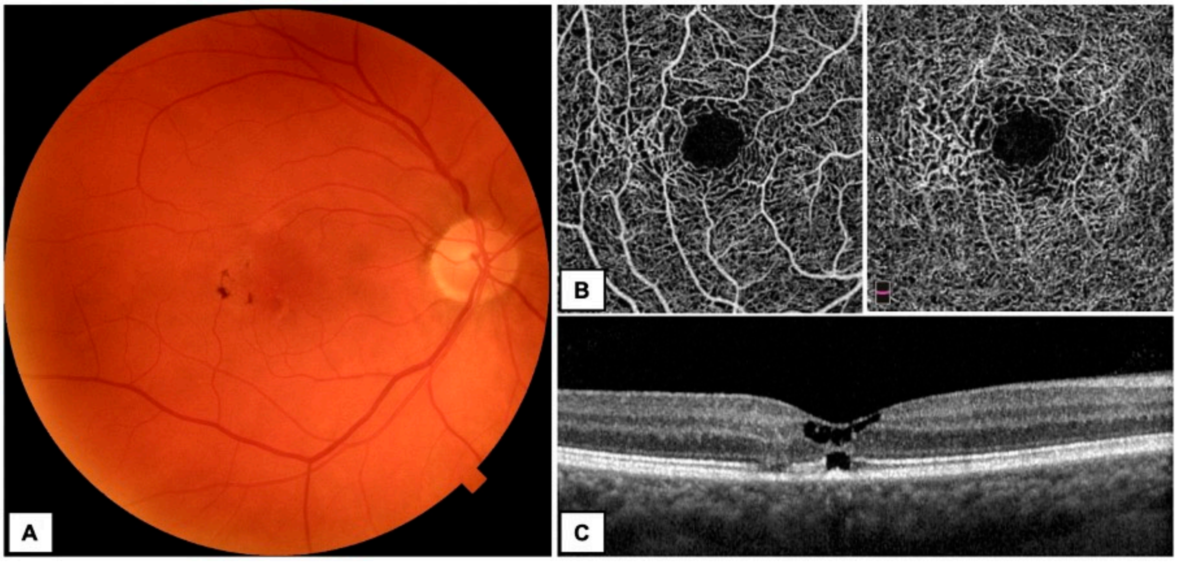

Figure S1. Mac Tel 2 of the RE (**A**) Color fundus photography–parafoveal loss of retinal transparency with RPE plaques temporal to the fovea. (**B**) OCTA–telangiectatic and distorted vessels both in the deep and superficial capillary plexuses mainly located temporal to the fovea (**C**) OCT B-scan shows intraretinal cavities in the inner retinal layers, disruption of the photoreceptors integrity in the center and temporal to the fovea.
